# Prevalence and risk factors of posttraumatic stress disorder among survivors five years after the “Wenchuan” earthquake in China

**DOI:** 10.1186/s12955-015-0247-z

**Published:** 2015-06-04

**Authors:** Li-Ping Zhang, Qian Zhao, Zhong-Cheng Luo, Yi-Xiong Lei, Yu Wang, Pei-Xi Wang

**Affiliations:** Medical school, Panzhihua University, Panzhihua, China; Institute of Public Health, School of Nursing , Henan University, Jinming Campus, Kaifeng, HN 475004 China; School of Public Health, Guangzhou Medical University, Guangzhou, China; Ministry of Education-Shanghai Key Laboratory of Children’s Environmental Health, Xin Hua Hospital, Shanghai Jiao-Tong University School of Medicine, Shanghai, China

**Keywords:** Posttraumatic stress disorder, Survivors, Risk factors, Wenchuan earthquake

## Abstract

**Objective:**

The aim of this study was to investigate the prevalence of posttraumatic stress disorder (PTSD) and its risk factors among survivors in a heavily-hit area five years after the Wenchuan earthquake in 2008, China.

**Methods:**

684 survivors from Beichuan county, the center of the Wenchuan Earthquake in 2008, were evaluated using the PTSD Checklist-Civilian Version (PCL-C) questionnaire in 2013.

**Results:**

The prevalence of PTSD among survivors was 9.2% in 2013. Significant risk factors of PTSD included gender (females 12.1%, males 5.2%), age (18–35 y 0.8%, 36–59 y 9.7%, ≥60 y 12.9%), occupation (farmers 12.2%, non-farmers 1.6%), education (less than high school 11.0%; > = high school 0.8%) and family member loss (yes: 12.4%, no: 7.3%). Multivariate logistic regression showed that females, older people, farmers and those with family member loss were significantly more likely to develop PTSD.

**Conclusions:**

Posttraumatic stress symptoms remained relatively common among survivors five years after the “5.12” Earthquake in Beichuan county, China. It is important to provide psychological aid and social support for survivors to decease health burden from PTSD, especially for females, farmers, old age survivors and those with family member loss.

## Background

On May 12, 2008, a major earthquake measuring 8.0 on the Richter scale hit Wenchuan country, Sichuan province of Southwest China. The “5.12” Wenchuan earthquake was the most violent earthquake in China since Tangshan earthquake measuring 7.8 on the Richter scale in 1976. During the earthquake, 69,227 people were dead and 374,643 got injured. The total property damages cost over 150 billion dollars [1].

Many studies have detected a variety of psychological problems following the “5.12” Wenchuan earthquake, especially posttraumatic stress disorder (PTSD) [2-4]. PTSD usually occurs one month after a disaster and could cause a series of psychological and physiological problems including intrusive re-experiencing, avoidance, numbness and hyper-arousal [5].

When a large scale disaster such as earthquake happens, usually PTSD would also develop [6-8]. About two thirds PTSD patients could recover over time, while others would become more serious, and the posttraumatic state could continue further to affect their cognitive and behavior functions for several years or even longer [9].

Previous studies have shown that PTSD is a common problem of mental health among survivors after natural disasters. According to a study about the Taiwan 9.21 earthquake, the PTSD rate was 20.9% among survivors who experienced the disaster 2 years after the earthquake [10]. The PTSD rate was 23% among orphans thirty years after the Tangshan earthquake in 1976, China [9]. For the Morocco earthquake, 10% of survivors developed posttraumatic stress symptoms forty years after the earthquake [11].

In this study, we assessed the prevalence and risk factors of PTSD among survivors five years after the Wenchuan earthquake. The prevalence of PTSD for survivors from the area of Beichuan was 12% about 2 years after the disaster in 2010 [12]. Continuing long-term monitoring of PTSD rates among survivors after the disaster could provide important information for implementing necessary community-based psychological aids in disaster areas. Five years after the disaster is an important time window in that subjects who remained to be suffered from PTSD after 5-y may need stronger psychological aid and treatment to recover. However, there is a lack of data on posttraumatic stress symptoms among survivors five years after the Wenchuan earthquake. This study thus aimed to investigate the prevalence and risk factors of PTSD among survivors five years after the Wenchuan earthquake.

## Methods

### Participants

All participants were recruited from Leigu Town with about 7,700 people. Most people in this community were originally native inhabitants of Leigu Town, a subordinate town of Beichuan County, which is the epicenter of the earthquake where 80% of the houses collapsed and more than 1,000 people died. Because Leigu Town was a severely afflicted area in the earthquake, almost all survivors received psychological aids after the earthquake. The survivors were the target population in this study, and were sampled from all household units in this community. A method of systematic sampling was adopted according to household unit doorplate number, and 730 families were selected. The sampling ratio was about 30%. Only one participant within a family was recruited for interview to ensure independence of study subjects, which is usually the household host if available. The inclusion criteria are as: (1) willing to participate, over 18 years old, clear consciousness and without language disorders; (2) experienced the Wenchuan earthquake in Beichuan county on May 12, 2008. The exclusion criteria include: (1) mental retardation, dementia, or any other major psychosis (e.g., schizophrenia, major depressive disorders, and severe mental disorders); (2) Failure to be reached after three household visits or refuse to be interviewed. If a sampled individual was unavailable, the next available individual was sampled. We have inquired the participant about the mental health before the earthquake. If they had similar symptoms such as depression, anxiety or another psychological problem, they would be excluded.

Since 2010, Community Health Service Center in China has set up a health care information system with data on all residents with major psychosis in the network of basic community public health care centers. The community health care information system maintains a list of residents with mental retardation, dementia, or any other major psychosis diagnosed by community doctors using questionnaires such as Mini-Mental State Examination (MMSE) and the General Health Questionnaire (GHQ-12). According to the list from the community health care information system, residents with mental retardation, dementia, or any other major psychosis were excluded.

### Procedures

The study was approved by the research ethics committee of Panzhihua University in Feb, 2013(reference number: 2013026). Written informed consent was obtained from all study participants. More than 20 voluntary individuals who can speak local dialect received training by the study investigators on communication and interviewing skills before the data collection. They were divided into six groups and all interview groups have been provided a professional physician. At the same time, we paid a return visit by random sampling to monitor the volunteers [13]. Interviewers visited the study community for data collection over 1 month between July 15 and August 15, 2013.

There were about 2300 families and 7700 people in this community. Systematic sampling by house doorplate number in three was conducted, and 730 families were selected. For the majority of families, there are three or more family members. Only one eligible resident in each family was recruited, usually a household host if available. We have chosen to recruit the first host we met for each family. If all family members were absent for one month or refused to be interviewed, the family with the next doorplate number would be selected as a replacement. If families with 2 or 3 consecutive doorplate numbers, all did not respond, the next ten doorplate numbers would be considered for recruitment. A total of 26 families refused to be interviewed, 20 families were not reachable within 1 month and 11 people that were unavailable because of mental problem. The final study cohort included 684 participants from Beichuan County who were representative of survivors in the heavily hit area.

All participants were interviewed using a structured interview questionnaire to obtain data on socio-demographic characteristics (age, gender, marital status, education, occupation) and earthquake related losses (property damage, personal injury, family member death). Posttraumatic stress symptoms were assessed using the subscale of PTSD Checklist-Civilian Version (PCL-C), which is the internationally recognized screen tool for PTSD with high reliability and validity [14,15]. The instrument is a self-reported 17-item symptom scale that corresponds to DSM-IV criteria [15]. The diagnosis of PTSD was determined by a clinical psychologist using the Chinese version of PCL-C [14,16,17] with Cronbach alpha coefficient of 0.82 and split-half reliability of 0.65 [16].

The 17 items of PCL-C each is with a score from 1 to 5: 1 = not at all; 2 = a little; 3 = quite a bit; 4 = extremely; 5 = very extremely. The maximal total score for 17 items is 85. Participant with a score of 50 or greater was classified as having probable PTSD. With PCL-C score of 50 as the diagnostic cut-off, the sensitivity was approximately 80% [18]. This cut-off point was used in the present study to identify PTSD cases.

According to the 4th edition of Diagnostic and Statistical Manual of Mental Disorder (DSM-IV) proposed by the Harvard Refugee Trauma Group [19], PTSD includes three groups of symptoms: B (intrusive re-experiencing, 5 items), C (avoidance or numbness, 7 items) and D (hyper-arousal, 5 items). Symptoms B positive group requires at least one of the five intrusive re-experiencing items to get a score of over 3. Symptoms C positive group requires at least three of the seven avoidance and numbness items to get a score of over 3. Symptoms D positive group requires at least two of the five hyper-arousal items to get a score of over 3 [19]. Cronbach’s alpha coefficient is 0.84. In this study, we did not use DSSM-IV criteria but used PCL-C score of 50 as the diagnostic cut-off. We compared the frequency of different PTSD symptoms (B, C, D) among all subjects as well as probable PTSD sufferers.

### Statistical analysis

All data were analyzed by statistical software SPSS 17.0 (SPSS, Chicago, Illinois, USA). Descriptive statistics were computed for demographic characteristic variables. Chi-square test was used to examine differences in categorical variables between those who were suffered from PTSD and those who were not. Multivariate logistic regression was used to assess the risk factors of PTSD.

## Results

Table 1 shows the main characteristics of participants. Males comprised 42.1% of the study sample. The average age was 51.8 years. About 37.3% of participants were > =60 years of age and 71.9% were farmers. A total of 37.7% of participants had family member loss in the earthquake.

**Table 1:** **Participants’ characteristics in the study cohort (N = 684)**

**Variables**		**n**	**%**
Gender			
	Male	288	42.1
	Female	396	57.9
Marital status			
	Single/divorced/widowed	176	25.7
	Married	508	74.3
Age (years)			
	18-35	129	18.8
	36-59	300	43.9
	≥60	255	37.3
Education level			
	Less than high school	566	82.8
	High school	66	9.6
	More than high school	52	7.6
Occupation			
	Farmer	492	71.9
	employee	95	13.9
	Self-employed	55	8.1
	Student	42	6.1
Ethnicity			
	Han	377	55.1
	Qiang (ethnic minority)	307	44.9
Property loss			
	Less(<10000 RMB/ 6135 USD)	110	16.1
	Large(> = 10000 RMB/6135 USD)	574	83.9
Loss			
	Family member died	258	37.7
	Family member injured	82	12.0
	Injury to self	25	3.7
	No death, no injury	319	46.6

As recommended by DSM-IV, total score over 50 was considered probable PTSD. The percentages of probable cases according to participants’ characteristics are shown in Table 2. A total of 63 participants (9.2%) had DSM-IV total score of over 50, and were identified as “probable PTSD cases”. The average PCL-C score was 31.98 (standard deviation (SD) =12.07). The significant variables identified for PTSD were gender, age group, education level, occupation and family member loss (all p < 0.05).

**Table 2:** **Proportion of** PTSD by **participants’ characteristics**

**Variables**	**N. of participants**	**Probable PTSD cases**	**Rate (%)**	**p value**
Overall	684	63	9.2	
Gender				0.002*
Male	288	15	5.2	
Female	396	48	12.1	
Marital status				0.588
Single/divorced/widowed	176	18	10.2	
Married	508	45	8.9	
Age (years)				<0.001*
18-35	129	1	0.8	
36-59	300	29	9.7	
≥60	255	33	12.9	
Education level				0.001*
Less than high school	566	62	11.0	
> = High school	118	1	0.8	
Occupation				<0.001*
Farmer	492	60	12.2	
Non-farmer	192	3	1.6	
Ethnicity				0.209
Han	377	30	8.0	
Qiang	307	33	10.7	
Property loss				0.443
Less(<10000 RMB/ 6135 USD)	110	8	7.3	
Large(> = 10000 RMB/ 6135 USD)	574	55	9.6	
Family member died				0.025*
No	426	31	7.3	
Yes	258	32	12.4	
Family member injured				0.822
No	602	56	9.3	
Yes	82	7	8.5	
Injury to self				0.232
No	659	59	9.0	
Yes	25	4	16.0	

PTSD = posttraumatic stress disorder.

*P < = 0.05.

Table 3 shows the frequencies of different PTSD symptoms. Among all 684 participants, the positive rates were 24.4% for “intrusive re-experiencing” group (B group), 4.4% for “avoidance and numbing” group (C group), and 13.2% for “hyper-arousal” group (D group), respectively. In subgroups, for items “Recurrent and intrusive distressing recollections”, “Intense psychological distress to cues” and “physiological reactivity to cues” PTSD symptoms appeared relatively frequently (15.5%, 15.5% and 15.6% respectively), while for items “detachment or estrangement feelings” and “restricted range of affect” the symptoms occurred relatively infrequently (2.8% and 2.5% respectively). Among the 63 probable PTSD participants, the percentages of positive symptoms for “intrusive re-experiencing”, “avoidance and numbing” and “hyper-arousal” groups were 84.1%, 34.9% and 52.4% respectively. The positive rates for the 17 items concerning PTSD symptoms varied from 20.6% to 76.2%. The highest items included “Intense psychological distress to cues” (73.0%) and “physiological reactivity to cues” (76.2%). The lowest item was “detachment or estrangement feelings” (20.6%).

**Table 3:** **The frequency of different PTSD symptoms among all subjects (684) and probable PTSD sufferers (n = 63)**

**Variables**	**All n (%)**	**PTSD n (%)**
**B group**	167(24.4)	53 (84.1)
Intrusive re-experiencing (at least one required)		
• Recurrent and intrusive distressing recollections	106 (15.5)	36 (57.1)
• Recurrent distressing dreams	57 (8.3)	25 (39.7)
• Acting or feeling as if events recurring	81 (11.8)	40 (63.5)
• Intense psychological distress to cues	106 (15.5)	46 (73.0)
• Physiological reactivity to cues	107 (15.6)	48 (76.2)
**C group**	30 (4.4)	22 (34.9)
Avoidance and numbing (at least three required)		
• Avoidance of thoughts, feelings and conversations	54 (7.9)	32 (50.8)
• Avoidance of reminders	56 (8.2)	28 (44.4)
• Psychogenic amnesia	26 (3.8)	17 (27.0)
•Markedly diminished interest in significant activities	22 (3.2)	15 (23.8)
• Detachment or estrangement feelings	19(2.8)	13 (20.6)
• Restricted range of affect	17 (2.5)	15 (23.8)
• Sense of a foreshortened future	42 (6.1)	24 (38.1)
**D group**	90 (13.2)	33(52.4)
Hyper-arousal (at least two required)		
• Sleep difficulty	62 (9.1)	23 (36.5)
• Irritability or outbursts of anger	37 (5.4)	18 (28.6)
• Difficulty concentrating	34 (5.0)	19 (30.2)
• Hyper-vigilance	84 (12.3)	29 (46.0)
• Exaggerated startle response	81 (11.8)	30 (47.6)

PTSD = posttraumatic stress disorder.

In Table 2, we have find five significant variables identified for PTSD were gender, age group, education level, occupation and family member loss (p < 0.05). The second order interactions between those 5 variables were tested with the Logistic regression model and we did not find any significant interaction (p > 0.05). The significant risk factors of PTSD in a multivariate logistic regression model are shown in Table 4. Female gender (OR = 2.08), older age (36–59 y: OR = 7.52, ≥60 y: OR = 9.64, vs. <36 y), occupation of farmer (OR = 4.37) and family member loss (OR = 1.88) were associated with significantly elevated risks of PTSD.

**Table 4:** **Significant risk factors of posttraumatic stress disorder from multivariate stepwise logistic regression models**

**Variable**	**β**	**SE**	**OR**	**95% CI**	***P***
Gender					
Male			1.0		
Female	0.73	0.315	2.08	1.12-3.85	0.02
Age (years)					
18-35			1.0		
36-59	2.02	1.040	7.52	1.03-57.75	0.05
≥60	2.27	1.043	9.64	1.25-74.46	0.03
Occupation					
Non-farmer			1.0		
Farmer	1.48	0.617	4.37	1.30-14.66	0.02
Family member died					
No			1.0		
Yes	0.63	0.273	1.88	1.10- 3.21	0.02
Constant	−7.02	1.217	0.001		<0.001

Variables considered for inclusion in the regression model were gender, marital status, age, education level, occupation, ethnicity, property loss, family member died, family member injured, Injury to self.

Only significant predictor variable were included in the final model.

β = partial Regression Coefficient; SE = Standard Error; OR = Odds Ratio; CI = Confidence Interval.

## Discussion

According to previous studies of the Wenchuan earthquake, the PTSD rate among survivors was 45.5% two and a half months after the earthquake [4]. The prevalence of probable PTSD among adults was 37.8% three months after the earthquake in Leigu Town [3], and 13.4% among children one year after the earthquake [20]. The probable PTSD rate in a heavily-hit area three years after the quake was 11.2% [21]. In the current study, we identified that the probable PTSD rate in a heavily-hit area five years after the earthquake was 9.2%, only 2.0% lower than the rate three years after the earthquake. This prevalence of PTSD is much higher than that in the general Chinese population, which was 0.2% [22].

We noted that the prevalence of PTSD seemed to decrease over time, but at a relatively slow pace. The declining prevalence may be attributable to self-healing capabilities, social support and psychological aid. Social support and psychological aid are shown to be associated with the quality of life in earthquake survivors [23]. Since at five years after the earthquake, the prevalence rate remains as high as near 10%, appropriate psychological aids may be warranted.

Significant risk factors for PTSD five years after the earthquake were female gender, old age (≥60 year), farmer occupation and family member loss. These findings are plausible. Our finding that the prevalence of PTSD was significantly higher among females (12.1%) compared with males (5.2%) is consistent with previous findings [2-4,24]. It seems that women exposed to traumatic events are more likely to develop PTSD than men.

Farmers appeared to be more likely to be suffered from PTSD. The incomes of farmers are generally low. They perhaps had fewer resources in coping with the devastating stress from the loss of economic support and living means than people of other occupations. This may result in a higher PTSD rate in farmers.

Survivors with family member loss may undertake more negative trauma, and have more economic and psychological pressure, which may result in a higher prevalence rate of PTSD [25].

Among all 63 probable PTSD sufferers, the items “physiological reactivity to cues” (76.2%) and “intense psychological distress to cues” (73.0%) occurred most frequently, suggesting more mental interventions concerning these symptoms would be helpful. Additionally, the majority of common PTSD symptoms are different for probable PTSD sufferers and others, suggesting different psychological aid may be needed. Community-based mental health aid may be helpful [26].

This study has limitations to acknowledge. First, in the current study participants with a score of 50 or above were classified as having probable PTSD without a clinical interview. This diagnostic criterion is often used when a clinical interview is not feasible [27]. It is expected that this method may not have optimal sensitivity and specificity. Second, the study sample was from a single town in a heavily-hit earthquake area in China. The findings from the current analysis may not be applicable to scenarios of earthquake or other natural disasters in other regions.

## Conclusion

PTSD remains a common mental health problem among survivors at five years after the 5.12 Wenchuan Earthquake in China. The major risk factors for such persistent PTSD included female gender, farmer occupation, old age and family member loss. Implementing psychological counseling for women, farmers, old age survivors and those with family member loss would be critical in mitigating the long-term risk of persistent PTSD.
